# Use of Simulation Resources for Underrepresented in Medicine Youth Engagement: A National Survey of Academic Anesthesiology Programs With Specified Diversity, Equity, and Inclusion Positions

**DOI:** 10.7759/cureus.44064

**Published:** 2023-08-24

**Authors:** Jonathan Curley, Mandy Flores-Curley, Siny Tsang, Kamilla Esfahani

**Affiliations:** 1 Anesthesiology and Critical Care, University of Virginia, Charlottesville, USA; 2 Education, University of Virginia, Charlottesville, USA; 3 Anesthesiology, University of Virginia School of Medicine, Charlottesville, USA

**Keywords:** community service, admissions/selection/minority recruitment, simulation in medical education, medical education, diversity and inclusion, diversity and equity in medicine

## Abstract

Background

The utilization of simulation resources can be an effective strategy to offer early medical exposure to underrepresented in medicine (URiM) youth populations, with the objective of promoting diversity in the field of medicine. Currently, it is unclear what proportion of academic anesthesiology programs with simulation centers utilize these resources for community engagement events.

Methodology

A survey was created using REDCap® and distributed via email to 38 anesthesiologists from 30 departments in the United States holding a leadership position dedicated to advancing diversity, equity, and inclusion. The survey assessed whether their programs had conducted community engagement events for URiM students, what simulation resources were available at their program, and which of these resources they had used at any community engagement events. Additionally, we assessed program characteristics such as region, academic versus community practice, and urban versus rural locations. Survey responses were collected between March and April 2023.

Results

We received responses from 15 of the 30 institutions sampled for an institutional response rate of 50%. The majority of respondents (86.7%) reported holding community engagement events. Most respondents reported a wide variety of simulation resources available, including 11 (73.3%) having access to full simulation centers. However, only three (27.3%) of the 11 with full simulation centers reported utilizing them for community events.

Conclusions

Despite the potential benefits of using simulation resources for community engagement events, our results suggest that academic anesthesiology departments may not commonly utilize simulation centers to provide URiM youth with exposure to the field of medicine. Anesthesiology departments with access to simulation resources are in a unique position to be leaders in advancing diversity in medicine by increasing URiM youth interest in medicine as a career through simulation-based exposure.

## Introduction

Demographic diversity in healthcare delivery organizations has been shown to improve patient care, workforce productivity, innovation, and financial stability [1]. To address the historic and persistent lack of diversity in medicine, the Liaison Committee on Medical Education mandates that medical schools develop diversity initiatives for both students and faculty [2]. Similarly, the Accreditation Council for Graduate Medical Education requires training programs to establish recruitment policies aimed at attracting underrepresented in medicine (URiM) populations for positions in residency and program leadership [3]. Despite these implemented standards, the proportion of URiMs in medicine remains significantly lower than their proportions in the general population [4-6].

Intentionally exposing URiM youth populations to a potential career in medicine can be an effective strategy to increase diversity in the field. Specifically, exposing URiM medical students to specific specialties increases their interest in pursuing those fields [7-9], while exposing URiM high school students to medicine raises their interest in pursuing this field [10,11]. Utilizing simulation for early exposure may be a practical strategy, as it eliminates the logistic barriers of in-person clinical exposure, allows for a controlled experience, and enables participants to perform engaging procedural tasks. Furthermore, simulation exposure has been shown to effectively increase medical student interest in exposed fields [12-14].

Perioperative educators, particularly anesthesiologists, with access to simulation resources, are well-suited to becoming leaders in implementing simulation-based community engagement with the aim of increasing interest in medicine among URiM youth populations. This study aimed to assess whether anesthesiology departments with specified diversity, equity, and inclusion (DEI) initiatives frequently utilize simulation resources for community engagement events and to advocate for such usage as a potential tool to increase diversity in medicine.

## Materials and methods

This cross-sectional, survey-based study was conducted with the approval of the Institutional Review Board for the Social and Behavioral Sciences (IRB-SBS) at the University of Virginia (UVA IRB-SBS #5764). As the survey was anonymous, participation was voluntary, and did not collect any identifying data, no individual participant consent was required.

An electronic survey was developed on the web platform Research Electronic Data Capture (REDCap®) [15,16] and comprised eight items (Appendices). The survey aimed to assess if academic anesthesiology departments with specified DEI initiatives commonly utilize simulation resources available for community engagement events. The face validity of all eight survey items was determined by the authors given they directly assessed our questions of interest. No survey pretesting or piloting was performed.

The first portion of the survey focused on community events and the age groups that were targeted at these events. The first item directly asked respondents if they were aware of their departments previously hosting community events with the specific aim of exposing low-income or minority school-aged or pre-medical college students to healthcare. Response options included “Yes” or “No.” Subsequently, respondents were asked to specify the age groups that had been targeted during these events. Respondents could select any and all listed response options, including college age, high school age, middle school age, elementary school age, or endorse that they were not aware of any such events.

The second portion of the survey assessed the availability of simulation resources at the respondent’s institution and whether these resources had been utilized for previously held community events. The third item of the survey asked respondents to identify the simulation equipment or resources available at their institution. Response options included medical equipment, available operating room space, airway manikins, ultrasound trainer, and fully interactive manikin/high-fidelity manikin (monitored, speech capable, and physical exam capable). In a separate item, respondents were prompted to specify which of these resources their departments had utilized for prior community events. Additionally, respondents were asked to provide a free text response describing any of the prior simulation community events they had been involved in. This final item of this portion was included to ensure the accuracy of the reported simulation use in the community events.

The final portion of the survey collected demographic data, including the geographic state of the respondents’ department and the categorization of their program as either academic or community-based, as well as urban large city, urban small/medium city, or rural. No other demographic data were obtained.

The survey was sent to 38 members of a collaborative DEI group called JEDI (Justice, Equity, Diversity, and Inclusion). This group consists of anesthesiologists with DEI leadership roles from 30 separate anesthesiology academic departments across the United States. The discrepancy of 38 members among 30 separate anesthesiologists was due to some departments having more than a single representative. The demographic data above provided a mechanism to identify any department with more than a single response. The survey was distributed via email with an embedded link, allowing only a single response per email address. The survey was available for completion from mid-March to mid-April, with a single reminder email sent midway through this period.

Statistical analysis

Responses were summarized into frequencies (n) and proportions (%). We examined the differences across categories using Fisher’s exact test in lieu of chi-square tests due to the small sample size. Statistical significance was set at an alpha value of 0.05. All statistical analyses were performed using R 4.1.2 [17].

## Results

A total of 15 responses were collected from the 38 surveys sent for an overall response rate of 39% and an institutional response rate from the 30 surveyed institutions of 50%. All 15 responses were from separate distinct anesthesiology departments, as determined by all respondents reporting their location from separate states. In total, 13 (86.7%) respondents reported holding community events, whereas two (13.3%) did not hold community events. Free text respondent event descriptions corroborated item check box descriptions of hosting events and the reported resources that were used. Most institutions reported having a variety of simulation resources available, including medical equipment (86.7%), operating room space (66.7%), airway manikins (86.7%), ultrasound (73.3%), high-fidelity manikins (66.7%), and a full simulation center (73.3%). As shown in Table 1, more than half of the institutions with airway manikins (84.6%), medical equipment (76.9%), or ultrasound (63.6%) reported utilizing them at community events. Although 11 (73.3%) institutions reported having full simulation centers, only three (27.3%) reported using the full simulation centers at community engagement events. Similarly, 10 (66.7%) reported having access to a high-fidelity manikin, yet only four (40%) reported using the high-fidelity manikin at community engagement events (Table 1).

**Table 1:** Frequencies and proportions of institutions with simulations available by utilization of the simulations.

		Simulation availability	
Simulation	Utilization	No	Yes
Medical equipment	No	2 (100%)	3 (23.1%)
	Yes	-	10 (76.9%)
OR space	No	5 (100%)	5 (50%)
	Yes	-	5 (50%)
Airway manikin	No	2 (100%)	2 (15.4%)
	Yes	-	11 (84.6%)
Ultrasound	No	3 (75%)	4 (36.4%)
	Yes	1 (25%)	7 (63.6%)
Interactive manikin	No	5 (100%)	6 (60%)
	Yes	-	4 (40%)
Full simulation center	No	4 (100%)	8 (72.7%)
	Yes	-	3 (27.3%)

Most institutions reported serving pre-medical undergraduate college students (86.7%) or middle school students (86.7%), with smaller proportions serving elementary school-aged students (73.3%) and high school students (66.7%). The majority of the respondents represented institutions in the Northeast (50%), with smaller proportions located in the West (21.4%), Midwest (14.3%), and South (14.3%). Most institutions were located in an urban large city (73.3%), with fewer in an urban small city (26.7%). With the exception that none of the institutions in the South (n = 2) served middle school students, there were no statistically significant differences in the geographic location or urbanicity of institutions between whether they served particular age groups (Table 2).

**Table 2:** Frequencies and proportions of institutions serving different age groups by geographic location and urbanicity. P-values reflect statistical significance from Fisher’s tests.

		Geographic location				
		Midwest	Northeast	South	West	P-value
Pre-medical	No	-	1 (50%)	1 (50%)	-	0.557
	Yes	2 (16.7%)	6 (50%)	1 (8.3%)	3 (25%)	
High school	No	1 (20%)	1 (20%)	2 (40%)	1 (20%)	0.188
	Yes	1 (11.1%)	6 (66.7%)	-	2 (22.2%)	
Middle school	No	-	-	2 (100%)	-	0.019
	Yes	2 (14.3%)	7 (50%)	-	3 (25%)	
Elementary school	No	1 (33.3%)	1 (33.3%)	1 (33.3%)	-	0.258
	Yes	1 (9.1%)	6 (54.5%)	1 (9.1%)	3 (27.3%)	
		Urbanicity				
		Urban large city	Urban small city			
Pre-medical	No	8 (80%)	2 (20%)			0.560
	Yes	3 (60%)	2 (40%)			
High school	No	4 (100%)	-			0.516
	Yes	7 (63.6%)	4 (36.4%)			
Middle school	No	8 (88.9%)	1 (11.1%)			0.235
	Yes	3 (50%)	3 (50%)			
Elementary school	No	10 (76.9%)	3 (23.1%)			0.467
	Yes	1 (50%)	1 (50%)			

## Discussion

Research has shown that exposing individuals to different career pathways can significantly increase their interest in pursuing those fields. This effect has been demonstrated among URiM populations, where exposure to specific fields has been found to increase interest in their corresponding areas, both among medical students and youth populations [7-11]. Additionally, simulation activities have been shown to be effective in generating interest in various career pathways [12-14]. Therefore, integrating simulation resources into community engagement events can serve as an effective strategy for fostering the interest of URiM youth populations in pursuing medicine as a career path.

Our survey revealed that within our small sample of anesthesiology departments with dedicated DEI initiatives, access to fully interactive simulation centers is common; however, departments do not often utilize these resources for community engagement events. The application of these findings to the entire field of anesthesiology is limited due to our decision to sample only a small cohort of programs. Nevertheless, our findings that even programs with defined DEI positions do not frequently utilize these resources for community events support the notion that this is not a common practice in the broader field of anesthesiology. Given that anesthesiology departments frequently have access to a wide variety of simulation resources, a notion supported by our findings, they are in a unique position to take a leadership role in promoting both community service and potentially diversity in medicine by increasing the use of such available simulation resources.

This survey-based study has numerous limitations that warrant further discussion. First, as previously mentioned, our study had a low sample size, with only 38 individuals from 30 different institutions being contacted. However, this study population was intentional, as we targeted academic anesthesiologists with known leadership roles in DEI initiatives based on the assumption that they would be more likely to be aware of any community outreach events compared to other members of their department. Additionally, our individual response rate was 39%, and our institutional response rate was 50% (all responses were from different institutions), which further decreased our final sample size. Although our department response rate of 50% was near to slightly above the average survey response rate of 44%, based on a meta-analysis of survey-based studies [18], the potential for a response bias remains. In addition, our decision to sample only a small proportion of all anesthesiology departments introduces the possibility of coverage error and limits our ability to apply our findings to all academic anesthesiology departments. However, our finding of a high self-reported lack of use of simulation for community events among leaders with dedicated DEI roles makes these biases less likely. Finally, our survey did not specify a time frame when inquiring about prior community events. Instead, respondents were simply asked if they were aware of any community events occurring. As a result, the responses may have been influenced by the time each individual respondent had been present at their current institution. Ultimately, these substantial limitations limit the validity of the proportions reported in our results, and these proportions should not be taken as accurate depictions of broad anesthesiology departmental practices. Instead, our findings highlight the possibility of utilizing simulation resources for community events and suggest that this practice may not be common.

Our findings, which suggest that simulation resources may not be commonly used by anesthesiology departments for community events, will hopefully encourage programs to leverage these resources for such purposes. Additionally, our findings may promote further research in this area. Specifically, further research in the form of case reports/series describing the implementation of such simulation events may be of value, as well as research providing outcome measurements to describe the effectiveness and impact of these events.

## Conclusions

Our survey-based study suggests that anesthesiology departments with dedicated DEI positions frequently engage in community engagement events, have a wide variety of simulation resources available, frequently have access to full simulation centers, yet infrequently utilize simulation centers for their community engagement events. We recommend that anesthesiology departments with access to simulation resources consider leveraging these resources for community events. In doing so, anesthesiology departments can serve their communities and promote medicine as a potential career path among low-income and URiM youth populations.

## Figures and Tables

**Figure 1:**
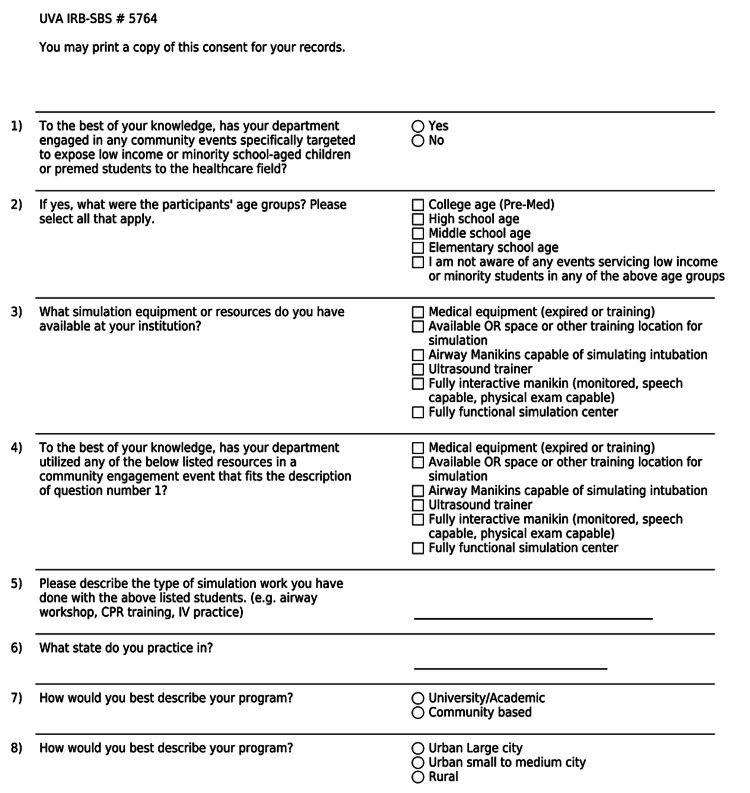
Study survey.
